# Depression, anxiety, stress, and fear of COVID-19 among Bangladeshi medical students during the first wave of the pandemic: a mixed-methods study

**DOI:** 10.3389/fpsyt.2023.1142724

**Published:** 2023-08-17

**Authors:** Md Ashiqur Rahman Ashiq, Pradip Sen Gupta, Md Abdullah Al Jubayer Biswas, Nowreen Ahmed, Mst. Sadia Sultana, Bikona Ghosh, M. Tasdik Hasan

**Affiliations:** ^1^Department of Public Health, American International University-Bangladesh, Dhaka, Bangladesh; ^2^Department of Epidemiology, Bangladesh University of Health Sciences, Dhaka, Bangladesh; ^3^Department of Statistics, University of Dhaka, Dhaka, Bangladesh; ^4^Department of Pharmacology, MH Samorita Medical College and Hospital, Dhaka, Bangladesh; ^5^Department of Public Health and Informatics, Jahangirnagar University, Savar, Dhaka, Bangladesh; ^6^Dhaka Medical College, Dhaka, Bangladesh; ^7^Action Lab, Department of Human-Centered Computing, Faculty of Information Technology, Monash University, Melbourne, VIC, Australia; ^8^Department of Public Health, State University of Bangladesh, Dhaka, Bangladesh; ^9^Public Health Foundation, Bangladesh (PHFBD), Dhaka, Bangladesh

**Keywords:** depression, anxiety, stress, fear, pandemic, medical students, Bangladesh

## Abstract

**Aim:**

This study aims to investigate depression, anxiety, stress, and fear of the COVID-19 pandemic and the associated risk factors among Bangladeshi medical students. It also explored qualitative insights on mental health from medical students during the first wave of the pandemic.

**Methods:**

This mixed-methods study was conducted online in Bangladesh from June 2020 to September 2020. Participants were Bangladeshi medical students from the first year to the final year. The quantitative part included a structured online survey. One focus group discussion (FGD) was organized using the Zoom platform to collect qualitative insights from the students. To determine levels of stress, anxiety, and depression, the Bangla-validated version of the *Depression, Anxiety, and Stress Scale 21 (DASS-21)* was used. A 7-item and Bangla-validated *Fear of COVID-19 Scale*, also known as *FCV-19S*, was used to explore the COVID-19-specific fear of the students. A semi-structured topic guide was used for exploring the qualitative insights of medical students' perceptions of fear of COVID-19, mental health impacts during COVID-19, overall recommendations to support students, and the impact of the pandemic on the future of the medical curriculum.

**Results:**

The study reported that 51.20%, 59.40%, and 64% of the 406 respondents had moderate to severe stress, anxiety, and depressive symptoms, respectively, according to the DASS-21. The mean fear score for the COVID-19 scale was 19.4 (SD 6.4). Respondents with family members aged 50 years or older (B = 2.1; CI: 0.3-3.9) and those who had infected family members (B = 1.9; 95% CI: 0.1-3.7) exhibited a higher level of fear of COVID-19. Moreover, depression was associated with a history of having cancer among family members (AOR = 2.9, CI: 1.1-7.5), anxiety was strongly associated with having symptoms of COVID-19 (AOR = 2, CI: 1.3-3.2), and stress was associated with having symptoms of COVID-19 infection among family members (AOR = 1.9, CI: 1.3-3). Altered sleep was a potential risk factor for developing stress, anxiety, and depression symptoms. Manual thematic analysis of qualitative data generated four major themes, including the perception of fear of COVID-19, the perception of mental health impacts during COVID-19, the change in the medical curriculum along with the pandemic, and recommendations from the medical students to support the mental health concerns of medical students during public health crises like this pandemic. Qualitative findings showed that the participants experienced fear of their parents becoming infected by COVID-19, and this fear was more prominent in those who had their loved ones hospitalized. They were also stressed and anxious, with thoughts of death. Their fear also extended to their thoughts on academic progress and the effectiveness of online classes.

**Conclusion:**

A substantial proportion of medical students experienced mental health difficulties in Bangladesh. Appropriate interventions should be designed, and adequate support should be provided to the medical students to protect their mental health and wellbeing, considering their potential impact on the future health system in a low-resource setting like Bangladesh.

## 1. Introduction

In the last 2 years, COVID-19 has spread worldwide, creating a global atmosphere of fear, anxiety, and uncertainty (1, 2). The relatively higher mortality has led the global scientific community to focus on prompt research for the development of treatments, vaccines, and preventive strategies, which has succeeded in containing the pandemic to some extent (3). Regardless of all the efforts, the pandemic contributed to the development of adverse mental health symptoms among the global population due to social isolation, financial stresses, academic uncertainty, an overload of information from the media, and the panic buying of daily necessities (4, 5). In Bangladesh, the number of infections and deaths was high, which created fear and mental health difficulties with social and economic ramifications among the residents (6).

All over the world, medical education is considered inherently stressful and demanding (7). In Bangladesh, undergraduate medical education (MBBS course) is divided into four phases (5 years in total): the first phase (Anatomy, Physiology, and Biochemistry) with a duration of one and a half years, the second phase (Pharmacology and Therapeutics and Forensic Medicine and Toxicology) with a duration of 1 year, the third phase (Community Medicine and Public Health, Pathology, and Microbiology) with a duration of 1 year, and the fourth phase (Medicine and Allied subjects, Surgery and Allied subjects, and Obstetrics and Gynecology) with a duration of one and a half years. In the second phase, students are exposed to small group teaching, clinical teaching, and formative assessment in Medicine and Allied subjects, Surgery and Allied subjects, and Obstetrics and Gynecology. There are in-course/formative (item/card/term) and end-course/summative (professional) exams in all four phases (8). This 5-year course is followed by a 12-month-long clinical internship to receive full registration as an MBBS doctor from the Bangladesh Medical and Dental Council (9). The continuous pressure of learning and mastering a huge amount of knowledge and skills compels medical students to sacrifice their personal and social lives to maintain moderate academic results in a competitive environment. This may lead to serious sleep deprivation, impaired judgment, reduced concentration, loss of self-esteem, and mental health issues such as increased anxiety and depression (10). Medical students have been found to have a higher prevalence of mental health disorders than the general public, including generalized anxiety disorder, depression, and burnout, even before the COVID-19 pandemic (11). A study conducted in Bangladesh before the pandemic showed that 33.5% of medical students had poor mental health status, with 38.9% experiencing depression and 17.6% having suicidal tendencies (12). Prior studies highlighted the significant psychological impact of the COVID-19 pandemic on medical students' mental health, including depression, anxiety, stress, and sleep disturbances (13–16). In addition, many medical students committed suicide (17, 18), which indicates the existence of severe mental health problems among this important population that is supposed to take responsibility for the future healthcare system.

The SARS epidemic displayed increased psychological distress among medical students across many affected countries (19). The risk of infection, academic uncertainty, disrupted learning experiences, and concerns regarding their family members seriously affected the mental health of the medical students of Bangladesh during the COVID-19 pandemic. A previous study found that female medical students and those in their final year of education were more vulnerable to poor mental health during the pandemic (16). The concept that Bangladeshi medical students are experiencing dramatically higher rates of depressive disorders (49.9%) and anxiety (65.9%) amid the pandemic has already been documented (20). However, to the best of our knowledge, the fear of COVID-19 and stress have not been adequately reported among Bangladeshi medical students, which points to a knowledge gap in the existing limited literature. Moreover, no study is available reporting the qualitative insights of medical students on this concerning issue.

This study was conducted to assess the fear of COVID-19, anxiety, stress, and depression symptoms among medical students in Bangladesh, determine the prevalence and associated factors of mental health difficulties, and explore insights on mental health issues using a mixed-methods design.

## 2. Methods

### 2.1. Study design and participants

A mixed-methods study (quantitative and qualitative) design was adopted to assess the fear of COVID-19, depression symptoms, anxiety, and stress during the COVID-19 pandemic among medical students in Bangladesh. The quantitative part followed a cross-sectional study design using an online questionnaire. One FGD (Focus Group Discussion) was conducted for the qualitative part. For both quantitative and qualitative parts, the study population consisted of registered and current Bangladeshi medical students. The inclusion criteria included the following criteria: willingness to participate; access to the Internet; willingness to provide informed consent; age of more than 18 years; and the ability to understand the Bangla language. For the quantitative part, an online convenience sampling technique was chosen to meet the study's aims and identify and recruit appropriate participants. Considering the risky data collection inside the medical college setting (most medical colleges are attached to hospitals) during the pandemic, the online survey was posted on closed social media (Facebook) groups of registered medical students in Bangladesh, and an open request was placed by a team of investigators to complete the survey. Moreover, three student volunteers (medical students) from different medical institutions were recruited and trained to circulate the online survey links and details among their student networks. They regularly posted the circular in social media groups. They were trained to be inclusive, open and to circulate details of the survey periodically for maximum reach. The study included participants from 53 public and private medical colleges in Bangladesh. The quantitative part of the study was conducted following the *Checklist for Reporting Results of Internet ESurveys (CHERRIES)* guidelines (21).

For the qualitative part, respondents were selected purposefully. The researchers communicated with some medical students they knew and requested that they participate in the focus group discussion. No monetary compensation was provided to the participants. As mentioned before, three medical students from three different medical institutions supported the study as volunteers. While circulating the online survey links and details of the project among their student networks, they communicated with some medical students and connected them to the study's student researcher (MARA). MARA checked their eligibility and availability for the FGD and invited them to participate. Those who participated in the FGD also completed the online survey form, though it was not a mandatory requirement for inclusion in the FGD.

### 2.2. Procedure

Between June 2020 and September 2020, the online survey was administered to the participants. The sample size for the quantitative part was calculated using the one-proportion formula. During the duration of the study, there was no literature pertaining to the study's outcome variables. Due to the unavailability of literature when the study was conceptualized, we assumed that 50% of Bangladeshi medical students suffered from fear, depression, anxiety, and stress due to the COVID-19 pandemic. We estimated our sample size to be 403 based on a 95% confidence interval, 5% precision, and a 5% non-response rate. Finally, we included quantitative data from 406 participants in the analysis. While the study was conducted, the nationwide strict lockdown was gradually lifted in Bangladesh, and government/non-government/autonomous offices were opened to a limited extent (22). However, graduate-level educational institutions were still closed for in-person classes.

The data collection tool for the quantitative part, an online semi-structured questionnaire, was created with Google Forms. The questionnaire was divided into four sections, including (i) sociodemographic information, (ii) pandemic-related information, (iii) the DASS scale for determining stress, anxiety, and depression, and (iv) the Fear of COVID-19 scale for determining fear. The questionnaire was first translated into Bangla; then, two bilingual experts retranslated it into English. Pretesting was conducted to ensure that the questionnaire was consistent, and additional changes were made to eliminate bias. The connection to the survey was posted on various social media platforms (i.e., Facebook and WhatsApp). On the first page of the survey, an information sheet outlining the purpose and procedure, the advantages of participating, and the right to reject participation in the study was presented, along with an electronic consent form. The participants were told that their knowledge would only be used for the purpose of the study. Anonymity and confidentiality were guaranteed.

The interview guidelines for the *Focus Group Discussion (FGD)* were developed and then translated into Bangla. The Focus Group Discussion was conducted in Bangla over a Zoom video call, and the whole session was recorded, transcribed, and translated into English to further analyze the qualitative data. Electronic informed consent from the FGD participants was obtained during the Zoom meeting before the discussions started using a Google form.

All the procedures of this study complied with the Code of Ethics of the World Medical Association (Declaration of Helsinki) for any experiments involving humans. The study was reviewed and approved by the Department of Public Health at American International University-Bangladesh (AIUB). The collected data were stored on a secured cloud server, maintaining the anonymity of the participants.

### 2.3. Measure

#### 2.3.1. Sociodemographic measures

Sociodemographic information on sex, age, study year, family monthly income, residing with family or not, history of the disease, etc., were gathered. Moreover, respondents were asked if they have any family members over 50 years old, as the death rate among 50+ people was more than 20% in Bangladesh, constituting a risk group (23).

#### 2.3.2. Pandemic-related questions

Pandemic-related information was sought from the respondents through some close-ended questions, including (i) history of having been infected by COVID-19; (ii) history of having COVID-19 infection among family members and neighbors; (iii) the deaths of close relatives; (iv) having COVID-19 symptoms; (v) having COVID-19 symptoms among the family members; and (vi) changing sleep pattern. There were three options for changing sleep: as before, sleep duration increased, and sleep duration reduced.

#### 2.3.3. Stress, anxiety, and depression symptoms

The Bangla-validated version of the Depression, Anxiety, and Stress Scale 21 (DASS-21) (24, 25) was used to assess the prevalence of stress, anxiety, and depression among students. The scale contains 21 items divided equally, with seven items divided into three subscales of stress, anxiety, and depression. The total score from each subsection can range from normal to extremely severe (a higher score indicates severity). The students responded to the items on a 4-point Likert scale (0 = never a problem, 1 = sometimes a problem, 2 = often a problem, and 3 = almost always a problem). Scores of mild symptoms of depression, anxiety, and stress were considered cut-off values for data analysis.

#### 2.3.4. Fear of COVID-19

A 7-item and Bangla-validated Fear of COVID-19 Scale, also known as FCV-19S (6), was used to explore the COVID-19-specific fear of the students (26). The scale contained items such as “I am most afraid of Corona” and “My hands become clammy when I think about Corona”, for example. The scale functions as a 5-item Likert-type scale. The response options included “strongly disagree”, “disagree”, “neutral”, “agree”, and “strongly agree”. The score ranged from 7 to 35. The scale was previously used in Bangladesh (27). The Cronbach's alpha for the FCV-19S was 0.83 in the present study.

#### 2.3.5. Interview guidelines for FGD

The interview guideline was drafted with semi-structured questions to obtain insights into medical students' perceptions of fear of COVID-19, mental health impacts during COVID-19, overall recommendations to support students, and the impact of the pandemic on the medical curriculum.

### 2.4. Statistical analysis

The statistical analysis for the quantitative part was conducted using SPSS version 25. All participant quantitative data were summarized using descriptive statistics such as frequency, percentage, mean, standard deviation (SD), median, and percentile. The Shapiro-Wilk W and Shapiro-Fancia W tests were conducted to analyze the distribution of the fear score. Stepwise linear regression with backward selection was conducted to investigate the fear-influencing factors of the participants. First, we conducted a bivariate linear regression model between the fear scores of the participants and a single predictor variable. Bivariable analysis results were reported as crude/unadjusted betas with a 95% confidence interval and coefficient of determination (R^2^). Then, we incorporated into the multivariable model all the influential predictive variables that were significant at the 5% significance level and reported results as unadjusted betas with a 95% confidence interval and coefficient of determination (R^2^). Similarly, we used stepwise binary logistic regression with backward selection to examine the relationships between depression, anxiety, and stress and the participants' demographics. At the 5% significance level, we included influential predictive variables of depression, anxiety, and stress in the final multivariable stepwise binary logistic regression. The outcomes of the final multivariable binary logistic regression were reported as odds ratios with 95% confidence intervals. A *P* < 0.05 was considered statistically significant.

For the qualitative part, thematic analysis was conducted manually. The thematic analysis approach constantly moves back and forth between the entire data set, the coded extracts of data, and the analysis of the data being produced. The analysis was conducted by the student investigator (MARA) and the senior author (MTH) using an iterative procedure (28). The transcription and translation of the FGD were carefully checked by a member of the research team who was a native speaker to ensure meticulousness. Then, at a semantic level, the initial codes were identified to understand themes following a systematic approach. Codes were sorted to develop the initial themes. Then, the research team reviewed the initial themes, refined them, and named them. Identified themes were carefully discussed with the interviewers (MARA and MTH) to ensure that interpretation was appropriate to FGD participants' experiences and to ensure rigor within the analyzed data. Thus, the iterative process produced the final themes, and interpretations were conceptualized accordingly.

## 3. Results

### 3.1. Characteristics of the study participants

Responses came from 406 medical students (with a mean age of 22.2 ± 1.7 years) from all over Bangladesh. The majority of respondents were aged 20–22 years (53.7%), women (60.8%), from the nuclear family (86.9%), with a monthly family income of 45,001–60,000 BDT (29.3%), and currently staying with family (93.4%). Most of them had family members aged 50 years or older (86.2%), 19.2% of the respondents had asthma, and 74.4% had a history of high blood pressure among family members. Respondents were mostly not infected by COVID-19 (74.1%), had no family members infected by COVID-19 (65.5%), and had neighbors infected by COVID-19 (65.5%) (Table 1).

**Table 1:** Characteristics of the study participants, Bangladesh (*N* = 406).

**Variable**	**Percentage (number)**
**Age in year**	
Mean ± SD	22.2 ± 1.7
≤ 19	4.9 (20)
20–22	53.7 (218)
23–25	38.9 (158)
≥26	2.5 (10)
**Gender**	
Male	39.2 (159)
Female	60.8 (247)
**Current study year in medical college**	
1st	10.8 (44)
2nd	15.5 (63)
3rd	29.1 (118)
4th	21.2 (86)
5th	23.4 (95)
**Type of family**	
Nuclear family	86.9 (353)
Joint family	13.1 (53)
**Monthly family income (BDT** ^*^ **)**	
Median (25th percentile, 75th percentile)	60,000 (45,000, 100,000)
≤ 45,000	25.6 (104)
45,001–60,000	29.3 (119)
60,001–100,000	27.8 (113)
≥100,001	17.2 (70)
**Currently staying with family**	
Yes	93.4 (379)
No	6.7 (27)
**Presence of 50**+ **aged family member**	
Yes	86.2 (350)
No	13.8 (56)
**Presence of children in the family**	
Yes	27.8 (113)
No	72.2 (293)
**History of having diseases**	
Cancer	-
Organ transplantation	-
Kidney complications	-
Heart disease	-
Diabetes	-
High blood pressure	7.9 (32)
Asthma	19.2 (78)
**History of having diseases of any family member**	
Cancer	12.3 (50)
Organ transplantation	-
Kidney complications	17.0 (69)
Heart disease	37.2 (151)
Diabetes	72.2 (293)
High blood pressure	74.4 (302)
Asthma	30.5 (124)
**Infected by COVID-19**	
Yes	6.4 (26)
No	74.1 (301)
Not known	19.5 (79)
**Family member infected by COVID-19**	
Yes	17.2 (70)
No	65.5 (266)
Not known	17.2 (70)
**Neighbor infected by COVID-19**	
Yes	65.5 (266)
No	20.4 (83)
Not known	14.0 (57)
**Death of closed relatives**	
Yes	19.0 (77)
No	81.0 (329)
**Having symptoms of COVID-19 infection onset of the epidemic**	
Yes	38.7 (157)
No	61.3 (249)
**Having symptoms of COVID-19 infection of the family member**	
**onset of the epidemic**	
Yes	49.3 (200)
No	50.7 (206)
**Changing of sleep since COVID-19 infection**	
Same as before	32.3 (131)
Sleep duration increased	43.6 (177)
Sleep duration reduced	24.1 (98)

^*^1 (one) USD = 108.72 Bangladeshi Taka (BDT) as per July 13, 2023.

### 3.2. Association of the fear of COVID-19 with the characteristics of the study participants

According to the bivariable analysis, the fear of COVID-19 was significantly associated with female sex (B:1.5; 95% CI:0.2–2.8), with monthly family income (≥100,001 BDT) (B:2.2; 95% CI:0.1–4.3), having family members aged 50 years older (B:2.1; 95% CI:0.3–3.9), having children in the family (B:1.6; 95% CI:0.2–3), having family members infected by COVID-19 (B:1.9; 95% CI:0.1–3.7), and the deaths of close relatives (B:1.7; 95% CI:0.1–3.4). There was also a significant association between a score of fear of COVID-19 and a history of having symptoms of COVID-19 at the onset of the pandemic among students themselves (B: 1.9; 95% CI:0.6–3.2) and their family members (B: 1.4; 95% CI:0.2–2.7). Reduced sleep duration (B: 3.8; 95% CI: 2.1–5.6) was also found to be significantly associated with fear of COVID-19. Multivariable analysis showed a significant association between fear of COVID-19 and female participants (B:1.6; 95% CI: 0.3–2.8) (Table 2).

**Table 2:** Bivariable and multivariable analysis showing association between fear score of COVID-19 with characteristics of study participants.

**Variable**	**Bivariable analysis**			**Multivariable analysis**	
	**R^2^**	**Beta (95% CI)**	***p*-value**	**Beta (95% CI)**	***p*-value**
**Age in year**					
20–22	0.006	2.1 (−1.5–5.6)	0.250	-	-
23–25		1.4 (−2.2–4.9)	0.448	-	-
≥26		2.2 (−3.1–7.6)	0.407	-	-
≤ 19		Reference			
**Gender**					
Female	0.014	1.5 (0.2–2.8)	0.025	1.6 (0.3–2.8)	0.017
Male		Reference		Reference	
**Monthly family income (BDT)**					
40,001–60,000		0.7 (−1–2.4)	0.442	-	-
60,001–100,000	0.014	0 (−1.7–1.8)	0.968	-	-
≥100,001		2.2 (0.1–4.3)	0.042	-	-
≤ 40,000		Reference			
**Presence of 50**+ **aged family member**					
Yes	0.015	2.1 (0.3–3.9)	0.020	1.5 (−0.2–3.3)	0.084
No		Reference		Reference	
**Presence of children in the family**					
Yes	0.013	1.6 (0.2–3.0)	0.030	1.6 (0.2–3)	0.028
No		Reference		Reference	
**History of having diseases of any family member**					
**Cancer**					
Yes	0.001	0.4 (−1.9–2.7)	0.738	-	-
No		Reference		-	-
**Kidney complications**					
Yes	0.004	1.2 (−0.7–3.1)	0.210	-	-
No		Reference		-	-
**Diabetes**					
Yes	0.005	1.0 (−0.4–2.4)	0.174	-	-
No		Reference		-	-
**Family member infected by COVID-19**					
Yes	0.011	1.9 (0.1–3.7)	0.043	-	-
Not known		0.4 (−1.3–2.1)	0.660	-	-
No		Reference		-	-
**Death of closed relatives**					
Yes	0.012	1.7 (0.1–3.4)	0.037	-	-
No		Reference		-	-
**Having symptoms of COVID-19 infection onset of the epidemic**					
Yes	0.021	1.9 (0.6–3.2)	0.006	1.2 (−0.1–2.5)	0.068
No		Reference		Reference	
**Having symptoms of COVID-19 infection of the family member onset of the epidemic**					
Yes	0.014	1.4 (0.2–2.7)	0.028	-	-
No		Reference		-	-
**Changing of sleep since COVID-19 infection**					
Sleep increased	0.052	0.7 (−0.8–2.1)	0.355	0.6 (−0.8–2)	0.380
Sleep reduced		3.8 (2.1–5.6)	< 0.001	3.6 (1.8–5.4)	< 0.001
Same as before		Reference		Reference	

*p* < 0.05 is significant.

### 3.3. Association of depression, anxiety, and stress with the characteristics of the study participants

**Figure 1:**
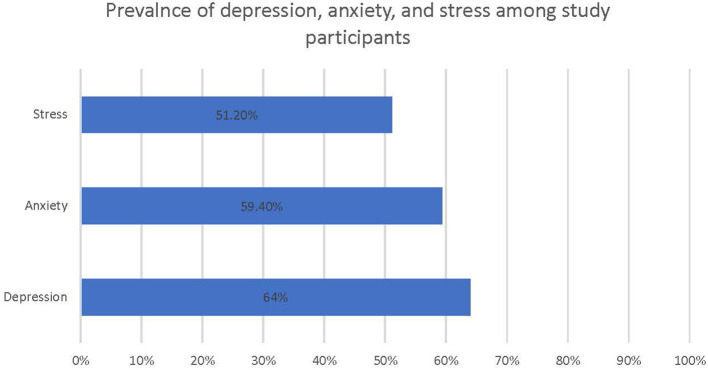
Prevalence of mild to severe depression, anxiety, and stress among study participants.

In the present study, 51.2% of the students reported suffering from anxiety, 59.4% from anxiety, and 64% from depression (Figure 1). Bivariable analysis showed that the score of the DASS stress subscale was significantly associated with the following factors: having a joint family, having family members aged 50 years or older, having children infected by COVID-19, and having family members infected by COVID-19 (Table 3). However, the multivariable analysis showed that the score of the DASS stress subscale was significantly associated with the monthly family income of 60,001–100,000 BDT (710–1,184 USD) (AOR-1.8; 95% CI:1–3.3), family members aged 50 years or older (AOR-2; 95% CI:1.1–4), children (AOR-1.7; 95% CI:1–2.8), a history of heart disease (AOR = 1.7; 95% CI:1.1–2.7), symptoms of COVID-19 among family members (AOR-1.9; 95% CI:1.3–3), and reduced sleep duration (AOR-4.3; 95% CI: 2.4–7.9).

**Table 3:** Bivariable analysis of association between score of DASS-21 with characteristics of study participants.

**Variable**	**Stress**		**Anxiety**		**Depression**	
	**OR (95% CI)**	***p*-value**	**OR (95% CI)**	***p*-value**	**OR (95% CI)**	***p*-value**
**Age in year**						
20–22	0.6 (0.2–1.5)	0.233	0.7 (0.2–1.8)	0.431	0.5 (0.2–1.5)	0.252
23–25	0.5 (0.2–1.4)	0.211	0.6 (0.2–1.5)	0.248	0.7 (0.2–1.9)	0.445
≥26	0.5 (0.1–2.5)	0.432	0.4 (0.1–2.1)	0.289	0.3 (0.1–1.7)	0.178
≤ 19	Reference		Reference		Reference	
**Type of family**						
Joint family	2.2 (1.2–4.1)	0.010	1.9 (1.0–3.5)	0.053	1.2 (0.7–2.3)	0.528
Nuclear family	Reference		Reference		Reference	
**Monthly family income (BDT)**						
40,001–60,000	1.3 (0.7–2.1)	0.392	1.1 (0.6–1.8)	0.809	1.5 (0.9–2.5)	0.157
60,001–100,000	1.9 (1.1–3.3)	0.018	1.3 (0.8–2.3)	0.283	2.2 (1.3–3.9)	0.005
≥100,001	2.4 (1.3–4.5)	0.006	2.1 (1.1–4.1)	0.021	2.9 (1.5–5.6)	0.002
≤ 40,000	Reference		Reference		Reference	
**Currently staying with family**						
Yes	1.9 (0.8–4.2)	0.132	1.2 (0.5–2.6)	0.677	2.4 (1.1–5.2)	0.032
No	Reference		Reference		Reference	
**Presence of 50**+ **aged family member**						
Yes	2.8 (1.5–5.1)	0.001	2.2 (1.2–3.9)	0.008	2.5 (1.4–4.5)	0.001
No	Reference		Reference		Reference	
**Presence of children in the family**						
Yes	1.7 (1.1–2.6)	0.026	1.7 (1.1–2.7)	0.026	1.7 (1.1–2.7)	0.027
No	Reference		Reference		Reference	
**History of having diseases**						
**High blood pressure**						
Yes	1.4 (0.7–3.0)	0.339	1.3 (0.6–2.9)	0.453	2.6 (1.0–6.5)	0.041
No	Reference		Reference		Reference	
**History of having diseases of any family member**						
**Cancer**						
Yes	2.8 (1.4–5.3)	0.002	2.1 (1.1–4.1)	0.027	4.8 (2.0–11.4)	0.001
No	Reference		Reference		Reference	
**Kidney complications**						
Yes	2.0 (1.2–3.4)	0.012	1.7 (1.0–3.0)	0.060	1.9 (1.1–3.4)	0.033
No	Reference		Reference		Reference	
**Heart disease**						
Yes	2.0 (1.4–3.1)	0.001	1.7 (1.1–2.6)	0.010	1.9 (1.2–2.9)	0.005
No	Reference		Reference		Reference	
**Diabetes**						
Yes	1.6 (1.1–2.5)	0.029	1.8 (1.2–2.8)	0.007	1.9 (1.2–3.0)	0.005
No	Reference		Reference		Reference	
**Infected by COVID-19**						
Yes	3.5 (1.4–8.9)	0.009	2.4 (0.9–6.1)	0.073	2 (0.8–5.2)	0.140
Not known	1.1 (0.7–1.9)	0.628	0.9 (0.6–1.6)	0.809	1.2 (0.7–2.1)	0.416
No	Reference		Reference		Reference	
**Family member infected by COVID-19**						
Yes	3.4 (1.9–6)	< 0.001	2.3 (1.3–4.1)	0.006	2.7 (1.5–5.0)	0.002
Not known	1.6 (0.9–2.7)	0.092	1.0 (0.6–1.8)	0.866	2.1 (1.2–3.8)	0.012
No	Reference		Reference		Reference	
**Neighbor infected by COVID-19**						
Yes	1.8 (1.1–3)	0.020	1.7 (1.1–2.8)	0.031	2.3 (1.4–3.8)	0.001
Not known	1.7 (0.9–3.3)	0.134	1.4 (0.7–2.8)	0.323	2.2 (1.1–4.3)	0.032
No	Reference		Reference		Reference	
**Death of closed relatives**						
Yes	1.6 (1–2.7)	0.057	1.8 (1.0–3.0)	0.034	1.8 (1–3.1)	0.044
No	Reference		Reference		Reference	
**Having symptoms of COVID-19 infection onset of the epidemic**						
Yes	2.2 (1.5–3.3)	< 0.001	2.2 (1.4–3.4)	< 0.001	2.4 (1.5–3.7)	< 0.001
No	Reference		Reference		Reference	
**Having symptoms of COVID-19 infection of the family member onset of the epidemic**						
Yes	2.5 (1.7–3.7)	< 0.001	2.0 (1.3–2.9)	0.001	2.1 (1.4–3.2)	0.001
No	Reference		Reference		Reference	
**Changing of sleep since COVID-19 infection**						
Sleep increased	1.8 (1.2–2.9)	0.009	1.7 (1.1–2.7)	0.023	2.2 (1.4–3.4)	0.001
Sleep reduced	4.7 (2.7–8.3)	< 0.001	5.1 (2.8–9.3)	< 0.001	4.5 (2.4–8.2)	< 0.001
Same as before	Reference		Reference		Reference	

*p* < 0.05 is significant.

In the bivariable analysis, the DASS score of the anxiety subscale was strongly associated with higher monthly family income (≥100,001 BDT compared to ≤ 40,000 BDT), family members aged 50 years or older, children, family members infected by COVID-19, and deaths of close relatives (Table 3). Moreover, the multivariable analysis displayed that the DASS score of the anxiety subscale was strongly associated with having children (AOR-1.8; 95% CI:1.1–3), a history of diabetes (AOR = 1.8, 95% CI: 1.1–2.9), the symptoms of COVID-19 (AOR-2; 95% CI:1.3–3.2), and decreased sleep duration (AOR-4.8; 95% CI:2.6–9).

It is worth mentioning that the DASS score of the depression subscale was reportedly associated with having family members aged 50 years or older, having children, family members infected by COVID-19, and the deaths of close relatives in the bivariable analysis. According to the results of the bivariable analysis, change in sleep since the COVID-19 infection and having high blood pressure were also reportedly associated with high DASS scores of depression (Table 3). Finally, the DASS score of the depression subscale was reportedly associated with having children (AOR-1.9; 95% CI: 1.1–3.2), a history of cancer among family members (AOR = 2.9, 95% CI: 1.1–7.5), and reduced sleep duration (AOR-4.1; 95% CI:2.1–7.9) in the multivariable analysis (Table 4).

**Table 4:** Multivariable analysis of association between score of DASS-21 and characteristics of study participants.

**Variable**	**Stress**		**Anxiety**		**Depression**	
	**AOR (95% CI)**	***p*-value**	**AOR (95% CI)**	***p*-value**	**AOR (95% CI)**	***p*-value**
**Monthly family income (Taka)**						
40,001–60,000	1.3 (0.8–2.4)	0.315	-	-	1.3 (0.7–2.4)	0.356
60,001–100,000	1.8 (1–3.3)	0.041	-	-	1.7 (0.9–3.1)	0.096
≥100,001	1.7 (0.9–3.3)	0.132	-	-	1.6 (0.8–3.4)	0.208
≤ 40,000	Reference		Reference		Reference	
**Currently staying with family**						
Yes	-	-	-	-	2.3 (0.9–5.7)	0.076
No	Reference		Reference		Reference	
**Presence of 50**+ **family member**						
Yes	2 (1.1–4)	0.034	-	-	-	-
No	Reference		Reference		Reference	
**Presence of children**						
Yes	1.7 (1–2.8)	0.032	1.8 (1.1–3.0)	0.017	1.9 (1.1–3.2)	0.018
No	Reference		Reference		Reference	
**History of having diseases of any family member**						
**Cancer**						
Yes	-	-	-	-	2.9 (1.1–7.5)	0.028
No	Reference		Reference		Reference	
**Heart disease**						
Yes	1.7 (1.1–2.7)	0.021	-	-	-	-
No	Reference		Reference		Reference	
**Diabetes**						
Yes	–	-	1.8 (1.1–2.9)	0.012	1.7 (1.1–2.8)	0.031
No	Reference		Reference		Reference	
**Family member infected by COVID-19**						
Yes	-	-	-	-	1.6 (0.8–3.3)	0.196
Not known	-	-	-	-	2.1 (1.1–4.3)	0.030
No	Reference		Reference		Reference	
**Having symptoms of COVID-19 infection onset of the epidemic**						
Yes	-		2.0 (1.3–3.2)	0.002	1.6 (0.9–2.8)	0.081
No	Reference		Reference		Reference	
**Having symptoms of COVID-19 infection of the family member onset of the epidemic**						
Yes	1.9 (1.3–3)	0.003	-	-	-	-
No	Reference		Reference	-	Reference	-
**Changing of sleep since COVID-19 infection**						
Sleep increased	1.9 (1.2–3.1)	0.012	1.8 (1.1–2.8)	0.020	2.1 (1.3–3.5)	0.003
Sleep reduced	4.3 (2.4–7.9)	< 0.001	4.8 (2.6–9)	< 0.001	4.1 (2.1–7.9)	< 0.001
Same as before	Reference		Reference		Reference	

*p* < 0.05 is significant.

### 3.4. Qualitative findings

The FGD included 10 medical students, with an equal distribution of five male and five female participants. The qualitative findings of this study generated four major themes, which included (i) perceptions of the fear of COVID-19, (ii) perceptions of mental health impacts during COVID-19, (iii) changes in the medical curriculum along with the pandemic, and (iv) recommendations from the medical students. Qualitative findings (thoughts, experiences, perceptions, and relevant recommendations) from the focus group discussion are presented here.

#### 3.4.1. Perception of the fear of COVID-19

During the initial days of COVID-19, most of the participants were not able to grasp the severity of the disease, and they perceived it as a vacation. However, over time, as the country's disease burden increased, fear gradually crept into their minds. Four participants expressed their concerns about the possibility of their parents being infected by COVID-19, which led to feelings of fear. According to a third-year medical student,

“*About 15 of my friends have either lost their parents due to COVID-19 or had to hospitalize them for being severely ill. Seeing these, I actually felt more afraid for the chances of my parents to be infected rather than mine”*

One of the participants who tested positive for COVID-19 added the following:

“*I think the fear has been extremely high among those who have seen near ones being hospitalized and fighting a battle with life”*

This idea was supported by another participant, who added that in the preliminary period when she was frightened about the pandemic and tried to maintain safety regulations, her parents rebuked her for being too cautious. Later, when her uncle and aunt contracted the infection, her parents became frightened and started to follow the rules and regulations.

However, one of the participants argued that the fear had significantly decreased among the common people over the past couple of months. He added that, as most of the people had already experienced this virus or had seen someone near them recover from it, they were not afraid of COVID-19 like before.

#### 3.4.2. Perception of mental health impacts during COVID-19

All of the respondents shared their thoughts on various mental health concerns. Despite being an outgoing person who engages in various extracurricular activities, one of the respondents resisted those activities and stayed at home. Even though she felt hopeless and frustrated sometimes, she could not take the risk of infecting her parents for the sake of her temporary relief.

During COVID-19, one participant felt worried as his father had faced a salary cut. Apart from the safety regulations and thoughts about the disease, they also had to cope with this scenario. Four of the participants expressed their anxiety regarding online classes. A final-year medical student was scared about his academic progress. Despite the teachers' cooperation, he found it difficult to gather clinical knowledge from online classes. He added the following:

“*I am even scared that if college opens now and we have to attend the exam on a short notice, will I be able to study and prepare myself like the way I did before?”*

Three other participants expressed their concerns about the current medical curriculum. They were mentally prepared for the possibility of graduating later than their friends from other disciplines. However, they felt anxious when they observed that most of their peers from previous batches had managed to graduate before the COVID-19 pandemic, and they were uncertain about when they would be able to take the final professional exams.

In this regard, another respondent added that she felt too stressed about fulfilling minor targets or had difficulties accomplishing minor deals. She further added the following:

“*(sic) Initially we were to attend an -hour online class every day, which is nothing compared to 8–10 h long classes we used to attend in normal times. But I felt so much pressure and anxiety to complete this task and had to struggle a lot to keep myself calm. This made me question my productive capability and I was further anxious.”*

One of the respondents shared that she felt overwhelmed and restless due to the posts on social media during this pandemic, where many of her friends used to post regularly about their achievements and success stories. The constant exposure to these posts motivated her to create her own success story, but she soon realized that she had no clear idea when her regular student life would resume. These thoughts weighed heavily on her, leading to a decline in her mental wellbeing.

Another respondent found himself becoming annoyed with everything and had repeated conflicts with his family members, which was quite the opposite of his regular nature. He also developed an avoidance tendency to finish chores like daily household activities, regular studies, or extracurricular tasks. Even though he was not a procrastinator, this was the first time in his life that he felt like running away from those chores. He also found these thoughts hindering his regular sleep cycle. This phenomenon is also supported by another participant, who stated the following:

“*Right now, I do not feel good in my home. (Sic) Neither am I enjoying a long sleep nor Facebook. I am not enjoying anything now”*

On a different note, one of the participants who tested positive for COVID-19, along with his family members, expressed that when they were hospitalized, he was deeply worried about his father, who is old. At that moment, he considered contemplating the mental health of his acquaintances as a luxury, despite acknowledging that he was experiencing tremendous stress and anxiety during those challenging times.

#### 3.4.3. Change in the medical curriculum

Many of the respondents worried that there was a lack of proper courses/guidelines on how to deal with such a pandemic in their curriculum. They suggested including a section in their curriculum about how to deal with or respond to pandemics or other public health disasters as medical students. Clear conceptions about the dos and don'ts will help them better prepare for the frontline in the future.

One enthusiastic participant also added this:

“*Medical students from 3rd year onwards can be trained for this kind of situation so that when there's a shortage of doctors or to help them during roster duties, students can come forward.”*

#### 3.4.4. Recommendations

The participants shared that medical students constantly face chronically enormous mental stress as they strive to balance their academic, personal, and social lives. Moreover, the ongoing pandemic added to their worries, affecting their present and future academic careers in many ways. They recommended a few points to support medical students' mental health concerns during this pandemic. One respondent mentioned feeling reassured and comforted if the teachers showed their concern for the students flexibly. He expressed the following sentiments:

“*If we know that they are going to have our back, it would be a big relief.”*

He further explained that a warm relationship between a teacher and their students could provide the passage for clear two-way communication. An empathetic approach toward other students can also play a vital role in reducing anxiety and stress among medical students.

Another participant stated that on social media, there was a constant unhealthy tension between the two groups. One group diligently adhered to safety measures and avoided social gatherings, while the other group resumed their pre-pandemic social lives as if everything was back to normal. Regarding this issue, one participant expressed the following sentiments:

“*If you think someone is not abiding by the rules and regulations of COVID-19, please don't attack him publicly. Rather talk to him in private and try to make him understand your views.”*

Another respondent shed light on the matter of expectations from family members placed upon medical students. He said that due to the challenges they face in their academics and concerns about their future career prospects, they might not be able to give it their best shot. Support from family members can help medical students in this regard.

From a very rational point of view, one respondent stated that the recent recruitment of doctors in government services would saturate the job market for some more years, which may lead to fewer vacancies in the post-COVID-19 scenario. He added that many medical students are depressed after seeing the deaths of many of their beloved teachers due to COVID-19 while fighting on the frontline. He urged that

“*Counseling sessions and psychotherapy must be arranged for helping us in academic and career prospects in order to tackle anxiety, fear and depression during this pandemic.”*

In summary, the qualitative findings reported that the participants experienced fear of their parents becoming infected by COVID-19, and fear was more prominent in those who had their loved ones hospitalized. They were also stressed and anxious with thoughts of death. Their fear also extended to their thoughts on academic progress, uncertainties, and the effectiveness of online classes.

## 4. Discussion

This study investigated the fear of COVID-19, stress, anxiety, and depression symptoms among Bangladeshi medical students. The mean score for the FCV-19 scale reported by this study was 19.4 (SD = 6.4), which is higher than the mean score for the FCV-19 scale (16.79, SD = 6.04) of a study conducted in Spain on university students (29). The chance of exposure to the virus can be one important difference between medical and university students. Another study from Serbia with a larger sample size (*n* = 1,722) conducted in May 2020 found the mean score for the FCV-19 scale to be 12.91 (SD = 4.5) (30). However, most of the Serbian medical students perceived the COVID-19 control measures as “good” or “very good.” Bangladeshi medical students in this study experienced lower stress (51.2%) than Peruvian medical students (65%) (31), Saudi Arabian medical students (55.2%) (32), and 54.5% among Irish medical students (33). All these studies used different tools to assess stress among medical students. However, Bangladeshi medical students exhibited higher levels of stress than those in France (36.83%) (34). By using the same tool (DASS 21), we found that Bangladeshi medical students were more stressed than Pakistani medical students (35). Additionally, using the same tool (DASS 21), we found that 59.4% of students suffered from anxiety in Bangladesh, which is higher than 44.5% among Turkish medical students (36), 57% among Peruvian medical students (31), 47.8% among Indonesian medical students (37), 52% among Pakistani medical students (35), 56.4% among Egyptian medical students (38), and 33.2% among medical students from Chennai, India (39). All of these studies were conducted during the first wave of COVID-19 in the respective countries. Except for India, more than 40% of the sample exhibited symptoms of anxiety.

A larger proportion of Bangladeshi medical students (64%) suffered from depression, where the prevalence of depression was 38.17% among French medical students (34), 62.7% among Pakistani medical students (39), 27.6% among Iranian medical students (40), 18.6% among Indonesian medical students (37), and 23.3% among Nepalese medical students (41). Even though the tools were different among the studies, Bangladeshi medical students displayed a higher level of anxiety symptoms when compared with their neighbors. It is worth noting that the rate of having symptoms of depression in medical students is higher than that of the general adult population in Bangladesh, which was found to be 36.57% in another Bangladeshi study (42). Considerably higher prevalence was observed among Turkish medical students (90.2%) (36), Egyptian medical students (75.2%) (38), and Peruvian medical students (74%) (31). The result of this study is somehow consistent with the findings of a previous Bangladeshi study conducted in the initial period of the COVID-19 pandemic among medical students, where 49.9% of them had depressive symptoms, and 65.9% had anxiety (20).

This study suggested that the fear of COVID-19 was strongly associated with the female sex, which is consistent with findings of research conducted earlier where females reported a higher level of fear of COVID-19 (43, 44). Surprisingly, the study showed that a higher monthly family income was significantly associated with a higher score of the fear of COVID-19. This finding is inconsistent with studies indicating that students with a higher ability to pay and a stable family income had less fear and lower psychological problems during this pandemic (45, 46). The fear of COVID-19 was also significantly associated with having children and family members aged 50 years or older as medical students. This finding might be because older family members are more vulnerable to COVID-19 infection, and younger generations experience greater fear of infecting their older family members (47).

The fear of COVID-19 was also significantly associated with having symptoms of COVID-19 among the medical students themselves, their family members, and infected family members. The deaths of close relatives had a positive association with fear as well. A study carried out in Bangladesh showed that the respondents who had suspected symptoms of COVID-19 had greater levels of stress than their counterparts (48). A tentative explanation could be due to the pandemic causing massive deaths. This might be the first time young people are exposed to the fear of the death of their family members so closely, which naturally generates a higher level of fear and anxiety (49). Corresponding to this study, a recent Bangladeshi study on students found that having suspected COVID-19 symptoms is significantly associated with the fear of COVID-19 (50). According to this study, reduced sleep was found to be associated with the fear of COVID-19. It was previously documented that fear and stress make one susceptible to psychological illness and insomnia (51). One tentative explanation could be that fear facilitates an adaptive response to fear through fear extinction, while sleep deprivation hampers the same process (52). It was documented before that ensuring a reliable source of health information can assist in elucidating fear (45).

Similar to the fear score, the DASS subscale of stress and depression symptoms was strongly associated with high monthly income, which is consistent with the findings of a previous study (53), but further research is required to understand the mechanism underlying it. However, other studies revealed opposite associations: stress, anxiety, and depression were associated with lower income (54–57). The DASS stress, anxiety, and depression subscales were positively associated with having children. This finding corresponds with a previous study suggesting that parenting stress is associated with anxiety and depression (58). A possible explanation might be that parents are the only reference point for the children during this home quarantine period and have to manage their work from home (59). Additionally, older members of the family usually suffer from different non-communicable diseases, which make them more prone to severe complications of COVID-19 (47); this might be a reason behind the higher levels of stress, anxiety, and depression among the respondents who had family members aged 50 years or older.

Having a history of different non-communicable diseases such as diabetes and heart disease was found to be associated with the fear of COVID as well as depression, anxiety, and stress. This might be due to several factors, including the cancellation of routine treatments, increased workload causing decreased staff availability for these treatment sessions, and reduced public transport for lockdown and social distancing measures (60). One of the important findings of this study is the notable association between experiencing symptoms of COVID-19, both among the participants themselves and among their family members, and the levels of stress and anxiety reported by the participants. Consistent with this finding, two previous Bangladeshi studies showed that having suspected COVID-19 symptoms was significantly associated with negative mental health consequences (61, 62). This finding might be attributable to the fact that respondents with suspected COVID-19 symptoms perceive those symptoms as COVID-19 (61, 62). In addition to those factors, such as being tested positive for COVID-19, family members were susceptible to depression. This is supported by another study conducted in Bangladesh, which indicated that participants who had COVID-19-infected relatives had higher rates of mental disorders (51). Additionally, anxiety and depression subscales were associated with the deaths of close relatives, which is in line with a previous study (51). The mechanism underlying this finding could be that individuals become more concerned about losing their loved ones (53).

Reduced sleep duration was also positively associated with stress, anxiety, and depression, which is supported by a study where 55.7% of the respondents were poor sleepers (63) and another one indicating that insomnia was associated with stress, anxiety, and depression (64). High levels of stress impair sleep quality, which could lead to sleep deprivation (65). Moreover, anxiety and depression could deteriorate sleep quality and increase the severity of insomnia, especially when both are present simultaneously (65). Increased sleep duration also had a significant association with stress, anxiety, and depression in the present study.

As medical students are the future of the health care system of a country, adequate measures should be taken to address their mental health issues. Periodic counseling sessions should be arranged for the medical students, and they must be encouraged by the medical college authorities to express their concerns and seek support rather than stigmatizing it. Accurate information sources should be ensured to elucidate fear. Moreover, specific clinical and counseling courses could be included in the curriculum of medical schools, which would assist medical students in fighting against negative mental health challenges from this pandemic and beyond.

The qualitative findings of this study have brought some key notions to light. The participants expressed their concerns regarding the health and safety of their parents. They had the tendency to follow the safety precautions strictly to prevent their parents from becoming infected. Moreover, the participants who were infected with COVID-19 expressed their fear, especially when they heard about death news frequently. However, this long lockdown made them think about various things, and some reported changing behaviors with more irritation and anxiety. Some students expressed concern over their academic progress. As the classes shifted online, students sometimes found it difficult to understand the clinical aspects of learning using technology. They also expressed concern about whether they would be good doctors through online classes or not. To cope with this pandemic, academic and career-related anxiety, and frustration due to a prolonged stay at home, the medical students suggested building up empathetic and student-friendly two-way communication so that the students could feel assured. The enthusiastic students also proposed to add a special section to their curriculum from where they can learn to fight any upcoming global health crisis like this pandemic. This study's findings clearly indicate that adequate mental health interventions and support programs targeting medical students are a critical need in Bangladesh. These findings call for providing low-intensity, regular, and specific institutional support to reduce fear of a pandemic or any health crisis, gender-sensitive interventions, grief management, training on managing sleep during crises and beyond, additional academic support, and peer support during changed scenarios like the COVID-19 pandemic. This pandemic pointed out a gap: our medical students are poorly managing their mental health during emergencies, possibly resulting from considering psychiatry as a less important academic entity. This is the time to keep psychiatry and mental health-related training at the center of medical education to support the growth of medical students as future physicians prioritizing their own mental health.

### 4.1. Strength and limitations

To the best of our knowledge, this is one of the first few studies to explore the fear of COVID-19 and several mental health symptoms among Bangladeshi medical students amid the pandemic. This study will assist in filling the knowledge gap in this less researched area and also help in designing appropriate interventions to safeguard medical students from negative psychological consequences during situations like the pandemic and other future public health crises. It also documented some important qualitative insights that are novel and important to understand the impact of the pandemic on their mental health.

However, this study has several limitations that need to be acknowledged. First, respondents were mostly of the higher socioeconomic group since Internet connection was mandatory. This limits the generalizability of the study's findings. Second, convenience sampling and self-reporting data might lead to selection and reporting biases. Third, considering its limited sample size, the study is not nationally representative. Finally, causal inferences cannot be elucidated due to the study's cross-sectional nature. Despite these limitations, the study's findings provide a wider outlook on the mental health aspect of the medical students of Bangladesh during the devastating pandemic and report useful insights to understand the contexts and future directives.

## 5. Conclusion

A substantial proportion of medical students are struggling with their mental health. Respondents with COVID-19-like symptoms, sleep disturbances, family members aged 50 years or older, infected family members, and those who experienced the death of relatives due to COVID-19 were at higher risk of developing mental health symptoms. Appropriate interventions should be designed, and adequate support should be provided, especially during critical periods like the pandemic, to the medical students to protect their mental health and wellbeing, considering the potential impact of them on the future health system in a low-resource setting like Bangladesh.

## Data availability statement

The raw data supporting the conclusions of this article will be made available by the authors, without undue reservation.

## Ethics statement

The studies involving human participants were reviewed and approved by Department of Public Health, American International University, Bangladesh. The patients/participants provided their written informed consent to participate in this study. Written informed consent was obtained from the individual(s) for the publication of any potentially identifiable images or data included in this article.

## Author contributions

MA: research design, data collection, data analysis, and manuscript writing. PG: research design, data interpretation, and manuscript writing. MJ: data analysis and data interpretation. NA: data collection, data analysis, and data interpretation. MS: data interpretation, manuscript writing, and validation. BG: manuscript writing and validation. MH: research design, data collection, data analysis, validation, and manuscript writing. All authors contributed to the article and approved the submitted version.
